# Design of a Fiber Bragg Grating Pressure Sensor Based on a Metal Diaphragm and Lever Structure

**DOI:** 10.3390/s22145096

**Published:** 2022-07-07

**Authors:** Zhaoyue Liu, Lina Zeng, Ke Xu, Zaijin Li, Hao Chen, Zhongliang Qiao, Yi Qu, Guojun Liu, Lin Li

**Affiliations:** Key Laboratory of Laser Technology and Optoelectronic Functional Materials of Hainan Province, College of Physics and Eletronic Engineering, Hainan Normal University, Haikou 571158, China; 20203085400243@hainnu.edu.cn (Z.L.); zenglina@hainnu.edu.cn (L.Z.); 20203085400244@hainnu.edu.cn (K.X.); 060115@hainnu.edu.cn (Z.L.); chenhao@hainnu.edu.cn (H.C.); qzhl060910@hainnu.edu.cn (Z.Q.); quyi@hainnu.edu.cn (Y.Q.); 068006@hainnu.edu.cn (G.L.)

**Keywords:** FBG, pressure sensing, elastic diaphragm, lever structure

## Abstract

In this paper, a pressure sensor based on a metal diaphragm and lever structure is designed, the sensing principle and mechanical structure of this sensor are analyzed and simulated, and its sensitization effectiveness and temperature compensation are verified. The maximum deflections of metal diaphragms of different sizes and materials were compared, and it was found that the square beryllium bronze diaphragm with a thickness of 1 mm and a side length of 20 mm had good elastic properties. The influence of the FBG in different positions of the lever on the center wavelength is analyzed. The sensitivity of the bare FBG is markedly improved under the influence of the two structures of the square elastic diaphragm and the lever, with a typical pressure sensitivity of 3.35 nm/MPa at 3 mm to the left of the lever center. The purpose of temperature compensation is achieved by adding another FBG that measures the temperature, and the sensing sensitivity can be tuned by adjusting the position of the FBG. It can meet the detection needs of a small range and high sensitivity.

## 1. Introduction

High-precision pressure measurement plays an important role in many application environments, such as marine monitoring, industrial pipeline measurement, oil prospecting, etc. However, traditional sensors based on electrical components have issues such as low stability, a slow response speed, and poor anti-electromagnetic interference ability. For example, although the thermal resistor has a high sensitivity, its stability is poor. The platinum resistance sensor performs better in terms of stability, but its response speed is not fast enough, and its sensitivity is not high, which affects the sensing effect of electrical components [1]. As an optical passive device, FBG has the advantages of a light weight, small size, anti-electromagnetic interference, pressure resistance and corrosion resistance, and network distribution [2,3]. It shows high sensitivity and stability even in complex application environments such as high temperatures and high pressures, narrow pipelines, the chemical industry, and industrial manufacturing [4,5,6,7,8,9,10,11].

Due to the low sensitivity of bare FBGs, most studies have used structures such as cantilevers [12,13], bellows [14], cylinders [15], and elastic diaphragms to sensitize FBGs. The elastic diaphragm structure has attracted more attention due to its advantages of a large range, high sensitivity, and easy series connection. The sensitizing materials used in the current research are usually carbon fiber materials [16,17], polymer materials [18,19,20,21,22], and metal materials. Among them, polymer materials are easily affected by temperature when used as pressure load diaphragms. Although carbon fiber materials are less affected by temperature and have high sensitivity, their impact resistance is low. Metal materials are less sensitive to temperature than polymer materials and have high mechanical strength. They are also suitable for simultaneous changes in temperature and depth. Gautam Hegde et al. [23] used martensitic stainless steel as the material of the elastic diaphragm to make a temperature-compensated diaphragm-type fiber Bragg grating sensor, and the temperature-compensated FBG was bonded to the strain-free area of the same diaphragm. The experimental results obtained show that the pressure sensitivity of the sensor is 3.64 pm/bar. Wang Li et al. [24] used metallized optical fibers to make a circular diaphragm type ocean temperature and depth sensor. The team focused on the resolution of the sensor resolution and accuracy, among which the accuracy of temperature depth reaches 0.1%, but no temperature compensation means are added. Qinggeng Fan et al. [25] proposed and studied a pressure sensor using a composite structure consisting of square diaphragms, steel trusses, and vertical beams. The sensor showed low hysteresis, and the pressure sensitivity was 622.71 pm/MPa. Subsequently, the team [26] designed a pressure sensor based on a single square diaphragm, which showed a relatively high temperature compensation effect, but the sensitivity was low. While the sensitization structures in the above research activities ensured high sensitivity, the size design is large, and it is difficult to reduce hysteresis or ensure high sensitivity for ensuring a better temperature compensation effect. In this regard, finding a simple structure that has better pressure sensitivity and can eliminate temperature compensation is the focus of research.

In this paper, a pressure sensing structure composed of a metal elastic diaphragm and a lever is proposed. Two FBG structures are used, one for pressure sensing and the other for temperature compensation. For temperature compensation, the pressure sensitivity is improved compared to the above studies, and the sensing sensitivity can be tuned by adjusting the position of the lever. In addition, the sensing principle of this structure and the mechanical structures of different metal diaphragms are analyzed and simulated, and the sensing effectiveness is verified.

## 2. Structural Design and Principle

**Figure 1:**
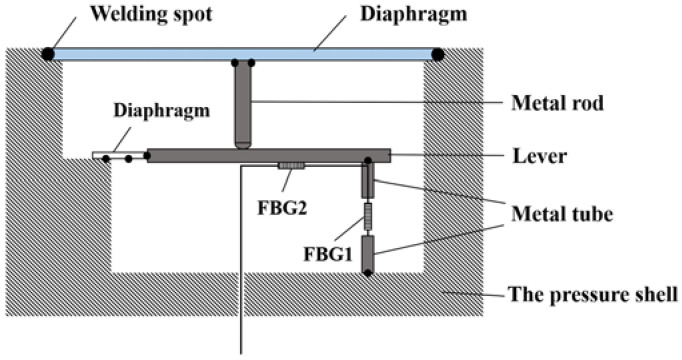
Schematic diagram of the pressure sensing structure.

The proposed pressure sensing structure is shown in Figure 1, which consists of two elastic diaphragms, a metal protective shell, a metal rod, two metal cylinders, a lever, and two fiber gratings connected in a series. The periphery of the elastic diaphragm is welded with the surface of the metal shell, the external pressure directly acts on the metal diaphragm, the deformation of the center of the metal diaphragm causes the connected metal rod to move downward, and the elastic diaphragm is connected by the elastic diaphragm. The lever is subjected to the pressure on the upper end, so the tail end of the lever presses down the FBG1, and the FBG2 is attached to the lever and is not affected by the pressure, which is used for temperature sensing and temperature compensation. Among them, applying prestress to FBG1 can expand the range of the sensor, but in the theoretical analysis, the applied prestress is ignored here. FBG1 is fixed in the upper metal cylinder with epoxy glue, and the final pigtail is also fixed by epoxy glue onto the metal shell.

### 2.1. Principle of Temperature Compensation

For FBG1, because it is affected by both external pressure and ambient temperature, the Bragg wavelength shift affected by both strain ε and temperature changes $\Delta T_{1}$ can be expressed as [15,16].

(1) $$\frac{\Delta \lambda_{B1}}{\lambda_{B1}}=\left(1-p_{e}\right)\varepsilon +\left(\alpha +\xi \right)\Delta T_{1}$$

where $P_{\varepsilon }$, α, and ξ are the effective photoelastic coefficient, thermal expansion coefficient, and thermo-optic coefficient of the fiber, respectively. Since FBG2 on the lever surface is only temperature-sensitive, the Bragg wavelength shift can be written as:

(2) $$\frac{\Delta \lambda_{B2}}{\lambda_{B2}}=\left(\alpha +\xi \right)\Delta T_{2}$$

Since these two FBGs are closely spaced, it is assumed that they are subjected to the same temperature change, i.e., $\Delta T_{1}=\Delta T_{2}=T$. Equations (1) and (2) can be simplified to:

(3) $$\Delta \lambda_{B1}=S\Delta P+S_{T1}\Delta T;\;\Delta \lambda_{B2}=S_{T2}\Delta T$$

where S and $S_{T1}$ are the pressure sensitivity and temperature sensitivity of FBG1, respectively. $S_{T2}$ represents the temperature sensitivity of FBG2. Therefore, by equation substitution to Equation (3), we get:

(4) $$\Delta \lambda_{B1}=S\Delta P+\frac{S_{T1}\Delta \lambda_{B2}}{ST_{2}}$$

It can be seen from Equation (4) that the temperature-induced pressure measurement error is eliminated and that temperature compensation is achieved.

### 2.2. The Principle of Pressure Sensing

**Figure 2:**
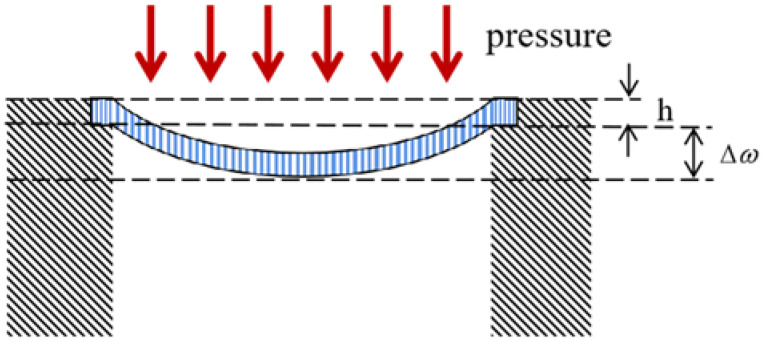
Schematic diagram of the diaphragm deformation and size.

The schematic diagram of the deformation of the diaphragm under pressure in the sensing structure is shown in Figure 2, in which the elastic diaphragm is deformed under the pressure load above. h is the thickness of the diaphragm and Δω is the displacement of the center of the diaphragm.

**Figure 3:**
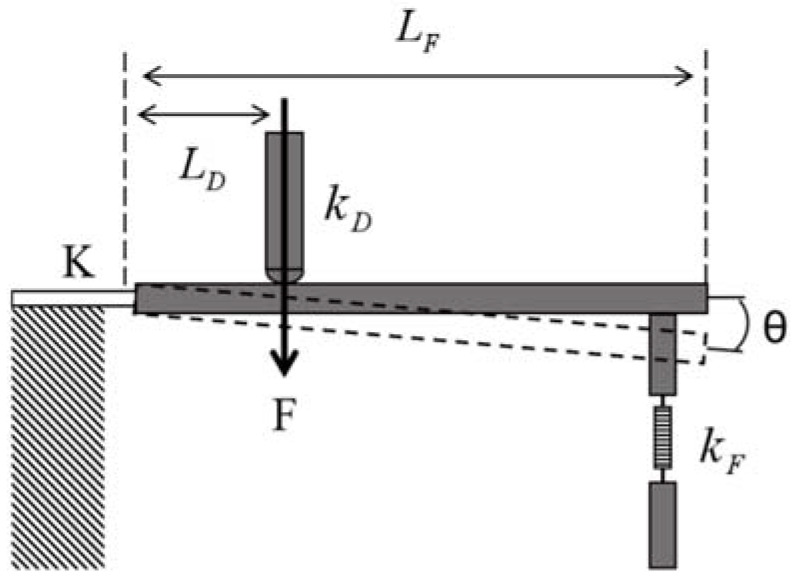
Schematic diagram of the mechanical model of the lever system.

When the metal diaphragm is deformed by the pressure load, it will drive the displacement of the metal rod fixed under the diaphragm. The schematic diagram of the mechanical model of the lever system composed of the metal rod, the elastic diaphragm, and the metal cylinder above FBG1 is shown in Figure 3.

The pressure at the center of the diaphragm drives FBG1 to stretch axially through the lever to achieve torque balance:

(5) $$FL_{D}-K\theta -k_{F}\Delta l_{F}L_{F}-k_{D}\Delta l_{D}L_{D}=0$$

In Equation (5), *F* is the axial pressure generated by the elastic diaphragm driving the metal rod, $\Delta l_{D}$ and $\Delta l_{F}$ are the displacement of the metal rod and the axial elongation of FBG1, respectively, $k_{D}$ and $k_{F}$ are the equivalent elastic coefficients of the elastic diaphragm and FBG1, respectively, *K* is the rotational stiffness of the plate, and θ is the angle at which the lever rotates. According to the geometric relationship,

(6) $$\Delta l_{D}=L_{D}\theta ,\Delta l_{F}=L_{F}\theta$$

Then, the shape variable of FBG1 caused by the metal bar is

(7) $$\Delta l_{F}=\frac{FL_{D}{}^{2}-K\Delta l_{D}-k_{D}\Delta l_{D}L_{D}{}^{2}}{k_{F}L_{F}L_{D}}=\varepsilon$$

Assuming that the temperature of the pressure sensor is constant, i.e., ΔT=0, the central wavelength offset of FBG1 is:

(8) $$\Delta \lambda_{B1}=\lambda_{B1}\left(1-p_{e}\right)\frac{FL_{D}{}^{2}-K\Delta l_{D}-k_{D}\Delta l_{D}L_{D}{}^{2}}{k_{F}L_{F}L_{D}}$$

Now, the metal diaphragm of different sizes and materials is simulated and analyzed to choose the appropriate elastic diaphragm to achieve a better sensing effect.

## 3. Simulation and Analysis

### 3.1. Analysis of the Metal Film Sheet

First, the shape and size of the diaphragm are analyzed, where the circular diaphragm and the square diaphragm are compared. The diameter of the diaphragm is 2*a*, the thickness is *h*, and the shape variable under the external pressure is Δω, where the shape variable of the square diaphragm is $\Delta \omega_{1}$ [17].

(9) $$\Delta \omega_{1}=\frac{12\left(1-\mu^{2}\right)}{47}\frac{a^{4}}{Eh^{3}}P$$

where *E*, *h*, and *μ* are the Young’s modulus, thickness, and Poisson ratio of the material, respectively. The form variable of the circular diaphragm is $\Delta \omega_{2}$.

(10) $$\Delta \omega_{2}=\frac{3\left(1-\mu^{2}\right)}{16}\frac{a^{4}}{Eh^{3}}P$$

**Figure 4:**
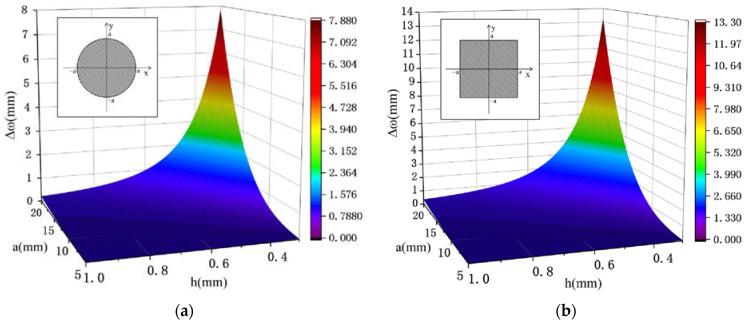
Simulation results of the central deflection vs. thickness h for the circular and square diaphragms. (**a**) Circular diaphragm size and its variation of center deflection. (**b**) square diaphragm size and its variation of center deflection.

At a boundary load of 1 MPa, the Young’s modulus was taken as 1.24 × 10^5^ MPa, the Poisson ratio was 0.35, and the radius and thickness of the circular and square diaphragm and their central deflection are shown in Figure 4.

As can be seen from Figure 4, the displacement of the center of the square of the same thickness increases with the increase in the elastic diaphragm edge length and the decrease in the thickness, which means that the square diaphragm is more sensitive to the change in pressure compared with the circular diaphragm. To achieve higher sensitivity, a square elastic diaphragm will be used here.

The following materials selected for the square diaphragm are analyzed. Here, spring steel, beryllium bronze, and ductile iron are selected for comparison. The specific parameters of the three materials are shown in Table 1.

**Figure 5:**
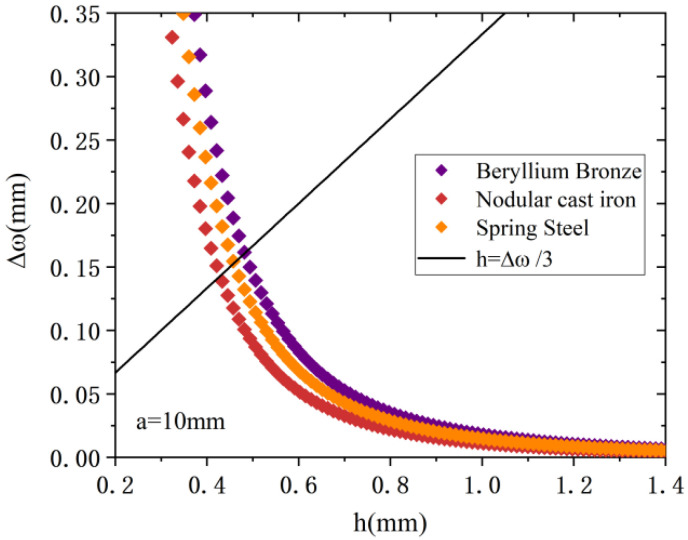
Plot of the square diaphragm Δω with a and h.

It is known from Equation (10) that materials with a small Young’s modulus and Poisson ratio can have a higher deflection. From the Small Deflection theory, in order to reduce the elastic hysteresis effect of the metal diaphragm, the maximum deflection of the square diaphragm is required to be less than one-third of its thickness. From Figure 4b, it is found that the deflection of the diaphragm does not change much within the radius range of 5 mm to 15 mm, so a = 10 mm is chosen here, and the relationship between the deflection and the thickness is analyzed in detail. The three materials are analyzed here to obtain the relationship among Δω and a and h, as shown in Figure 5.

From Figure 5, at the radius of 10 mm, the elastic diaphragm with a thickness above 0.5 mm satisfies the theory of small deflection. Compared with the other two materials, beryllium bronze has a lower Poisson ratio and Young’s modulus. Under the same pressure load, its deflection changes most obviously, so it has the best elastic performance among the three metal diaphragms, Therefore, beryllium bronze will be selected as the elastic diaphragm material. In order to ensure the small size of the sensor and the large sensitivity, the beryllium bronze square diaphragm thickness was set at 1 mm.

**Figure 6:**
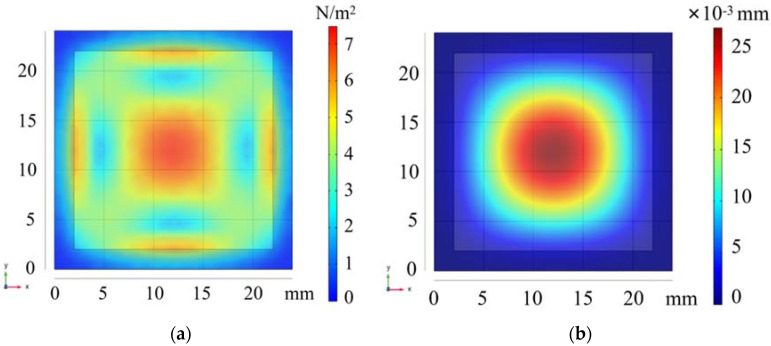
Distribution diagram of the force and shape variables of the square diaphragm. (**a**) Force distribution of square diaphragm. (**b**) Deformation variable distribution of square diaphragm.

Finite element analysis of the selected beryllium bronze square diaphragm and the force distribution and shape variables for the boundary load of 1 MPa are shown in Figure 6.

In Figure 6b, the displacement of its central point is 0.0268 mm, verifying the maximum deflection formula of the square diaphragm.

### 3.2. Sensing Analysis

**Figure 7:**
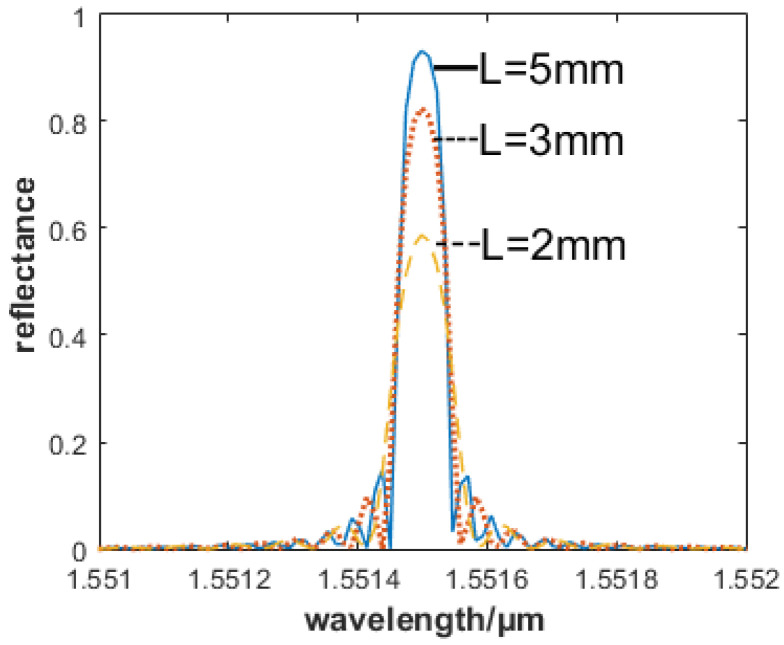
FBG reflectance and gate area length relationship.

First, the parameters of the selected FBG are analyzed. Here, the center wavelength of the FBG is set to 1551.15 nm. In the case of the same gate period and modulation intensity, the relationship between the reflectivity and FBG length is shown in Figure 7. From Figure 7, we know that the central reflectance can be obtained by selecting the FBG with the longer gate length and higher reflectance.

In order to ensure the overall size of the sensor, the FBG length x is set at 3 mm, the effective length L is 10 mm, and the spacing between FBG1 and FBG2 is 12 mm.

In the lever system, when the metal tube pressure is given to the elastic diaphragm, FBG1 is connected to the bottom of the lever. The FBG1 position $L_{F-R}$ directly affects the pressure on FBG. Considering that, with the increase in external pressure, the lever rotation angle increases with the displacement of the metal rod. There are two main reasons for this: (1) the larger the rotation angle, the larger the friction between the elements; (2) the lever will offset the axial stress of FBG1. Overall, a smaller boundary loading would improve the accuracy of this sensing structure. The following parameters are adopted in our experiment: the radius of the metal rod is 0.5 mm, the length is 3 mm, the size of the elastic diaphragm is 4 mm × 2 mm × 0.5 mm, and the length of participation in the deformation is 2 mm. The main parameters of the sensing structure are shown in Table 2.

**Figure 8:**
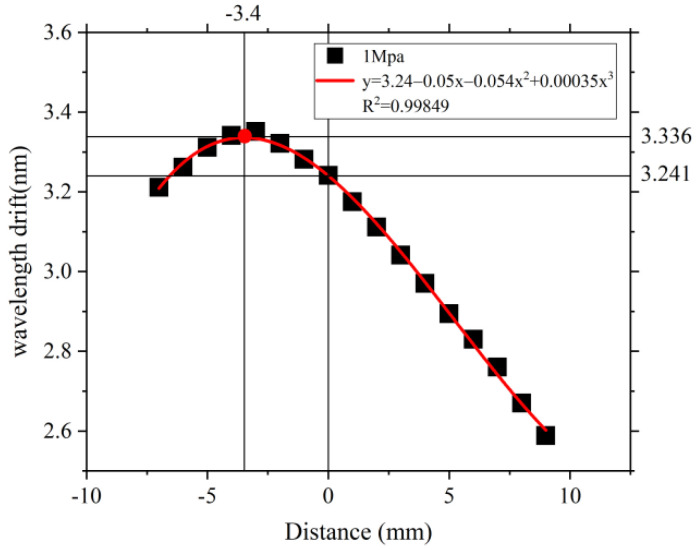
Effect of the FBG and metal bar distance on the wavelength change.

Here, the length of the lever and the strain produced by the FBG were analyzed under the square diaphragm boundary load of 1 MPa. By constructing the model and substituting the pressure value into Equation (10), the relationship between $L_{F-R}$ and $\Delta \lambda_{B}$ is shown in Figure 8.

In Figure 8, when the axial distance of the metal rod from the FBG1 is 0, the FBG1 is located just below the metal bar, it is equivalent to no leverage structure, and the central wavelength offset at this time is 3.241 nm. As the FBG1 moves along the lever towards the lever tail end, due to the presence of the rotational stiffness K of the elastic diaphragm, and as θ gets bigger, the effect of the boundary loading directly on FBG1 becomes smaller. Because of the leverage principle, FBG1 reduces the overall pressure sensitivity. As the FBG1 moves towards the elastic diaphragm, the largest shape variable is received at 3.4 mm away from the left side of the metal bar; at this point, the single elastic diaphragm amplifies the external pressure effect. Therefore, the feasibility of the proposed sensitization system is verified.

**Figure 9:**
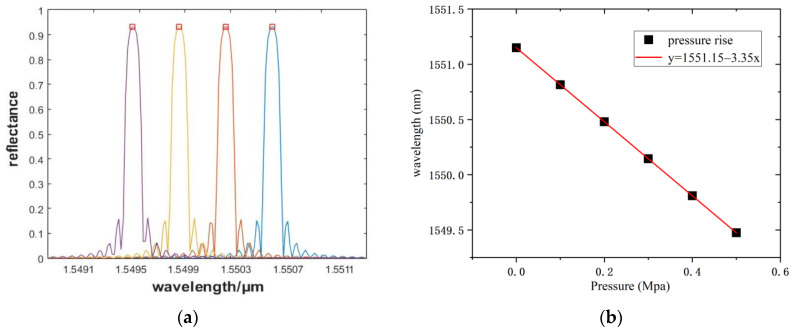
Central wavelength offset and its relation to pressure. (**a**) The variation of wavelength with increasing pressure. (**b**) Pressure sensitivity under 0−0.5MPa.

Considering the size of the device, a distance of 3 mm from the left of the metal rod was selected as the placement point of FBG1. Considering the different boundary loads for the square diaphragm from 0 MPa to 0.5 MPa, with a 0.1 MPa step, the shape variable is calculated by Equation (9), and the relationship between the central wavelength and the pressure change is obtained through Matlab, as shown in Figure 9.

In Figure 9a, the FBG reflection spectra of four of the different pressures are selected, and the center wavelength of FBG at 0–1 MPa is drawn as a diagram in Figure 9b. For the proposed pressure sensing structure, a pressure sensitivity of 3.35 nm/MPa can be obtained from the theoretical analysis and simulation. Considering the structure size of the sensor, the effective length of the FBG affects the pressure sensing range, so the sensor has a higher sensitivity in a small range.

## 4. Conclusions

This paper designed a pressure sensor based on a square diaphragm and lever structure. The sensing principle and mechanical structure have been analyzed and simulated, verifying its sensitization effectiveness and temperature compensation. The maximum deflection of square diaphragms of different sizes is analyzed. The deflections of the three metal diaphragms under the same pressure are compared. The results show that beryllium bronze has the best elastic performance among the three metal diaphragms, and the influence of FBG on the central wavelength at different positions of the lever is compared. Finally, the pressure sensitivity of 3.35 nm/MPa at 3 mm from the left side of the metal rod has been achieved, which can meet the detection requirements of liquid pressure in a shallow sea and fine pipeline. At present, the sensitivity of the FBG pressure sensor can be changed by adjusting the material and size of the elastic diaphragm. Generally speaking, the larger the deflection, the higher the sensitivity of the sensor. Adjusting the effective length of FBG can improve the range of the sensor to a certain extent, but it will reduce the sensitivity. Our follow up work will involve the system construction of this sensing structure, the pre-stretching analysis and pressure experiment of FBG, and the response time analysis of FBG1 and FBG2 in order to synchronize the temperature response of both and study the packaging process to obtain higher pressure sensitivity and sensing repeatability.

## Figures and Tables

**Table 1:** Parameters of the three materials.

Material	Spring Steel	Beryllium Bronze	Nodular Cast Iron
Young’s modulus/10^5^ MPa	2.06	1.24	1.59
Poisson ratio	0.3	0.35	0.28

**Table 2:** Main Parameters of the Pressure Sensing Structure.

Parameter	P/MPa	$p_{e}$	E/10^5^ MPa	μ	a/mm	h/mm	λ/nm	L/mm	x/mm	$L_{F}/\mathrm{mm}$
Value	1.0	0.22	1.24	0.35	10	0.5	1551.15	10	3	16
